# Risedronate does not reduce mechanical loading-related increases in cortical and trabecular bone mass in mice

**DOI:** 10.1016/j.bone.2011.03.775

**Published:** 2011-07

**Authors:** Toshihiro Sugiyama, Lee B. Meakin, Gabriel L. Galea, Brendan F. Jackson, Lance E. Lanyon, Frank H. Ebetino, R. Graham G. Russell, Joanna S. Price

**Affiliations:** a Department of Veterinary Basic Sciences, The Royal Veterinary College, University of London, London NW1 0TU, UK; b School of Veterinary Sciences, University of Bristol, Bristol BS40 5DU, UK; c Department of Veterinary Clinical Sciences, The Royal Veterinary College, University of London, Hertfordshire AL9 7TA, UK; d Warner Chilcott (Ireland) Ltd., Discovery, Research & Development, Dundalk, Ireland; e Nuffield Department of Orthopaedics, Rheumatology & Musculoskeletal Sciences, The Oxford University Institute of Musculoskeletal Sciences, The Botnar Research Centre, Nuffield Orthopaedic Centre, Oxford OX3 7LD, UK; f The Mellanby Centre for Bone Research, Department of Human Metabolism, The University of Sheffield Medical School, Sheffield S10 2RX, UK

**Keywords:** Bisphosphonate, Risedronate, Mechanical loading, Functional adaptation, Osteoporosis

## Abstract

To establish whether the combination of anti-resorptive therapy with mechanical loading has a negative, additive or synergistic effect on bone structure, we assessed the separate and combined effects of risedronate and non-invasive dynamic loading on trabecular and cortical bone. Seventeen-week-old female C57BL/6 mice were given daily subcutaneous injections of vehicle (n = 20) or risedronate at a dose of 0.15, 1.5, 15 or 150 μg/kg/day (n = 10 in each) for 17 days. From the fourth day of treatment, the right tibiae were subjected to a single period of axial loading (40 cycles/day) for three alternate days per week for two weeks. The left tibiae were used as internal controls. Trabecular and cortical sites in the tibiae were analyzed by high-resolution micro-computed tomography and imaging of fluorochrome labels. In the non-loaded tibiae, treatment with the higher doses of risedronate at 15 or 150 μg/kg/day resulted in higher trabecular bone volume and trabecular number than in vehicle-treated controls, whereas such treatment was associated with no differences in cortical bone volume at any dose. In the loaded tibiae, loading induced increases in trabecular and cortical bone volume compared with contra-lateral controls primarily through increased trabecular thickness and periosteal expansion, respectively, independently of risedronate treatment. In conclusion, the response to mechanical loading in both trabecular and cortical bone in mice is therefore not impaired by short-term treatment with risedronate, even over a 1000-fold dose range. In considering the optimization of treatments for osteoporosis, it is reassuring that anti-resorptive therapy and mechanical loading can exert independent beneficial effects.

**This article is part of a Special Issue entitled Bisphosphonates.**

## Research highlights

► We assessed the combined effect of a bisphosphonate and loading on bone structure. ► Non-invasive tibia loading and a 1000-fold dose range of risedronate were used. ► Loading significantly increased both cortical and trabecular bone mass. ► Risedronate does not reduce the osteogenic effect of loading.

## Introduction

Bisphosphonates play a central role in the management of osteoporosis [1–3]. Their major mechanism of action is to suppress osteoclast function and survival [4,5]. Due to the normal coupling of bone resorption to formation, one of their effects is to lower bone turnover [6]. Some of these drugs have also recently been demonstrated to protect osteocytes from apoptosis *in vivo* [7,8]. In contrast to the anti-resorptive effects of bisphosphonates, mechanical loading is the predominant functional osteogenic factor responsible for maintaining structurally appropriate levels of bone mass in adults [9,10].

By suppressing bone resorption, bisphosphonates effectively slow the decline in bone mass due to any cause including decreased mechanical loading [11–15]. The question remains as to their effect on the (re)modeling associated with a net osteogenic stimulus such as that derived from a therapeutic regimen of exercise. Some pilot clinical reports have shown an additive effect of bisphosphonates and exercise on areal bone mineral density [16,17], but other trials failed to find such an additive effect [18–20]. In experiments involving treadmill exercise in ovariectomized rats, the combination of etidronate, alendronate or risedronate treatment with exercise had additive or synergistic effects on bones [21–23], whereas zoledronic acid and exercise did not show either effect [24].

Since exercise would induce significant changes in cardio-pulmonary and nervous systems as well as skeletal muscle, the effect of combining bisphosphonates with local mechanical stimulation has been studied in a variety of rodent loading models. Again, however, the results are not consistent [25–28]. The effect of clodronate on periosteal apposition was increased when combined with mechanical loading in the rat tibia [25], whereas a recent study suggested that zoledronic acid impaired cortical bone's response to loading in the mouse tibia [28]. In contrast, alendronate, risedronate and zoledronic acid at clinical doses did not influence periosteal expansion induced by loading in the rat ulna [27]. Only one study investigated the effect of combining a bisphosphonate with loading in trabecular bone and showed that pamidronate did not change osteogenesis caused by invasive loading in the rat tail [26].

Among the currently available bisphosphonates with different mineral binding and biochemical actions, risedronate has a comparatively lower affinity for bone mineral [29,30], which may facilitate its access to the mechano-responsive cells of the osteocytic lacunar network [31]. Importantly, risedronate has a relatively potent action on the appendicular skeleton [4,32]. In the present study, we assessed the separate and combined effects of various doses of risedronate with external mechanical loading on trabecular and cortical bone, by using the non-invasive mouse tibia axial loading model [33,34]. This approach has the advantage that it allows examination of the effect of local mechanical stimulation, distinct from that of exercise, in both trabecular and cortical bone compartments.

## Materials and methods

### Animals

Virgin, female C57BL/6 mice were purchased from Charles River Laboratories Inc. (Margate, UK) at 7 weeks of age, and housed in sterilized polypropylene cages (n = 5 per cage) with free access to water and a maintenance diet containing 0.73% calcium, 0.52% phosphorus, and 3.5 IU/g vitamin D (RM1; Special Diet Services Ltd., Witham, UK) in a 12-hour light/dark cycle, with room temperature at 21 ± 2 °C. All procedures complied with the UK Animals (Scientific Procedures) Act 1986 and were reviewed and approved by the ethics committee of the Royal Veterinary College (London, UK).

### Experimental design

At 17 weeks of age, 60 mice were divided into five body weight-matched groups and treated with daily subcutaneous injections of vehicle (saline; n = 20) or risedronate (Procter & Gamble Pharmaceuticals, Inc., Mason, Ohio, USA) at a dose of 0.15 (n = 10), 1.5 (n = 10), 15 (n = 10) or 150 (n = 10) μg/kg/day for 17 days (days 1–17). 1.5 μg/kg/day is a dose equivalent to that used clinically in osteoporosis patients based on a mg/kg basis and on its known low intestinal absorption. During this treatment, the right tibiae were subjected to external loading under isoflurane-induced anesthesia for three alternate days per week (approximately 7 min/day) on days 4, 6, 8, 11, 13 and 15. Normal activity within the cages was allowed. The non-loaded contra-lateral (left) bones were used as internal controls, as has previously been validated in the model used in the present study [34] and confirmed by others in the rat ulna axial loading model [35]. High doses of calcein (50 mg/kg; Sigma Chemical Co., St. Louis, Missouri, USA) and alizarin (50 mg/kg; Sigma Chemical Co.) were injected intraperitoneally on the first and last days of loading (days 4 and 15), respectively. At 19 weeks of age (day 18), the mice were euthanized and their tibiae were collected for analysis. Body weight was measured before (day 1) and after (day 18) these treatments. Although it could have been potentially interesting to use ovariectomised animals [36,37], we chose to simplify the experimental design and to study a full dose response to risedronate in intact animals.

### *In vivo* external mechanical loading

The apparatus and protocol for non-invasively loading the mouse tibia have been reported previously [33,34,37–39]. In brief, the flexed knee and ankle joints are positioned in concave cups; the upper cup, into which the knee is positioned, is attached to the actuator arm of a servo-hydraulic loading machine (Model HC10; Zwick Testing Machines Ltd., Leominster, UK) and the lower cup to a dynamic load cell. The tibia is held in place by a low level of continuous static “pre-load”, onto which higher levels of intermittent “dynamic” load are superimposed. In the present study, 0.5 N was used as the static “pre-load” which was held for approximately 7 min. The 11.5 N of “dynamic” load was superimposed onto the 0.5 N static “pre-load” in a series of 40 trapezoidal-shaped pulses (0.025 s loading, 0.050 s hold at 12.0 N and 0.025 s unloading) with a 10 s rest interval between each pulse. Strain gages attached *ex vivo* to the proximal tibial shaft of similar 17-week-old female C57BL/6 mice showed that a peak load of 12.0 N engendered approximately 1200 microstrain in that region [38].

### High-resolution micro-computed tomography (μCT) analysis

**Fig. 1:**
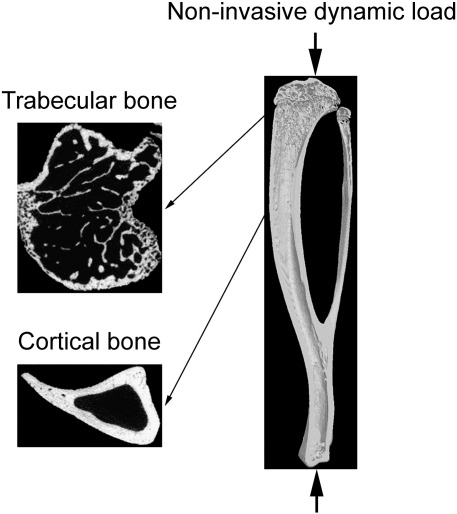
Direction of mechanical loading in the mouse tibia and transverse μCT images at the trabecular (0.25–0.75 mm distal to the growth plate) and cortical (0.5 mm long section at 37% of the bone's length from its proximal end) sites analyzed.

The tibiae were stored in 70% ethanol and scanned by μCT (SkyScan 1172; SkyScan, Kontich, Belgium) with a pixel size of 4.8 μm. The images of the bones were reconstructed using SkyScan software. As shown in Fig. 1, three-dimensional structural analyses were performed using SkyScan software for trabecular bone (secondary spongiosa; 0.25–0.75 mm distal to the growth plate) and cortical bone (0.5 mm long section at 37% of the bone's length from its proximal end). The parameters evaluated included bone volume/tissue volume (BV/TV), trabecular number and trabecular thickness in the trabecular region, and bone volume, periosteally enclosed volume and medullary volume in the cortical region. Since it has previously been shown that the primary effect of the present short-term loading model is increased osteogenesis [34,40], high-resolution μCT was selected to quantify functional adaptation. This method enables us to analyze precisely comparable sites of the loaded and contra-lateral control tibiae because the effects of loading are site-specific and the mouse bone is small.

### Calcein and alizarin labels imaging by confocal microscopy

After scanning by μCT, the bones were dehydrated and embedded in methyl methacrylate as previously described [34]. Transverse segments were obtained by cutting with an annular diamond saw. Images of calcein and alizarin labeled bone sections were visualized using the argon 488 nm laser and HeNe 543 nm laser, respectively, of a confocal laser scanning microscope (LSM 510; Carl Zeiss MicroImaging GmbH, Jena, Germany) at similar regions as the μCT analysis.

### Statistical analysis

All data are shown as mean ± SE. Body weight and lengths of the left control and right loaded tibiae were compared by one-way ANOVA. Mixed model analysis was performed on the six μCT parameters (trabecular BV/TV, trabecular number, trabecular thickness, cortical bone volume, periosteally enclosed volume and medullary volume). The model fixed effects were risedronate treatment (0, 0.15, 1.5, 15, 150 μg/kg/day) and mechanical loading (yes, no). Animal ID (n = 60) was included as a random variable to account for pairs of left and right tibiae belonging to the same mouse. Final body weight was included as a fixed covariate due to its influence on bone size (significant correlations between body weight and cortical μCT parameters of the left tibiae were confirmed in vehicle-treated mice). Post-hoc comparison following mixed model analysis was carried out using Bonferroni adjustment. Statistical analysis was performed using SPSS for Windows (version 17.0; SPSS Inc., Chicago, USA) and *p* < 0.05 was considered to be significant.

## Results

Initial and final body weight and longitudinal lengths of the left control and right loaded tibiae are shown in Table 1. There were no significant differences between the body weights or bone lengths of mice treated with vehicle or risedronate at any dose.

### Effects of risedronate alone

**Fig. 2:**
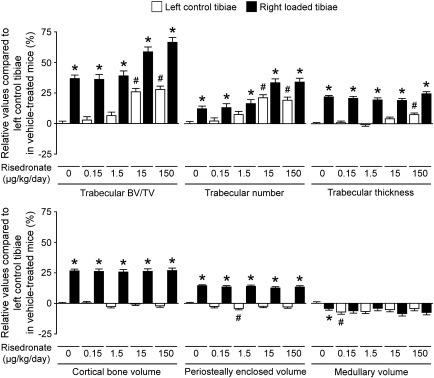
Relative values of trabecular and cortical μCT parameters of the left control and right loaded tibiae in mice treated with vehicle or risedronate at a dose of 0.15, 1.5, 15 or 150 μg/kg/day compared to the left control tibiae in vehicle-treated mice. Values were obtained from mixed model analysis including body weight and are presented as mean ± SE (n = 20 in vehicle treatment and n = 10 in risedronate treatments). ^#^*p* < 0.05 *versus* left control tibiae in vehicle-treated mice and *p < 0.05 *versus* left control tibiae in each treatment with vehicle or risedronate by mixed model analysis followed by Bonferroni adjustment.

In trabecular bone, treatment with risedronate at a dose of 15 or 150 μg/kg/day resulted in a significantly higher BV/TV of the left non-loaded tibiae than in vehicle-treated controls (Table 2, Fig. 2). This increase was primarily associated with higher trabecular number. In cortical bone, there were no significant differences in bone volume between vehicle-treated and risedronate-treated animals at any dose. A dose of 0.15 μg/kg/day induced a lower medullary volume than in vehicle-treated controls, while at a dose of 1.5 μg/kg/day there was a slightly lower periosteally enclosed volume (Table 2, Fig. 2).

### Effects of mechanical loading alone

As has been shown previously [34,37,38], mechanical loading significantly increased both trabecular BV/TV and cortical bone volume (Table 2, Fig. 2). The former effect was primarily due to an increase in trabecular thickness, while the latter response was mainly associated with an increase in periosteally enclosed volume.

### Combined effects of risedronate and mechanical loading

**Fig. 3:**
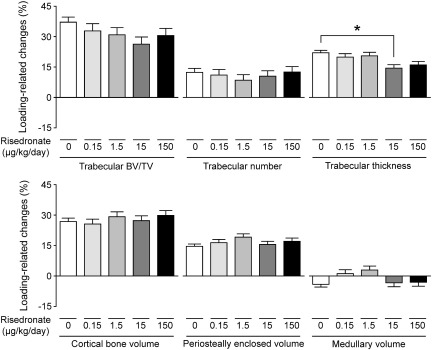
Loading-related changes ([right loaded − left control] / left control) in trabecular and cortical μCT parameters in mice treated with vehicle or risedronate at a dose of 0.15, 1.5, 15 or 150 μg/kg/day. Values were obtained from mixed model analysis including body weight and are presented as mean ± SE (n = 20 in vehicle treatment and n = 10 in risedronate treatments). *p < 0.05 by mixed model analysis followed by Bonferroni adjustment.

**Fig. 4:**
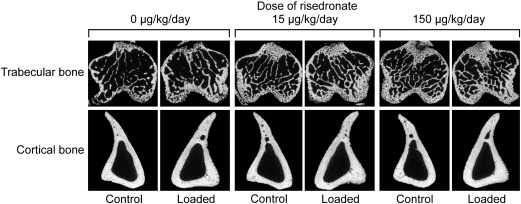
Representative transverse μCT images of the left control and right loaded trabecular (0.25 mm distal to the growth plate) and cortical (37% of the bone's length from its proximal end) bone in the tibiae of mice treated with vehicle or risedronate at a dose of 15 or 150 μg/kg/day.

**Fig. 5:**
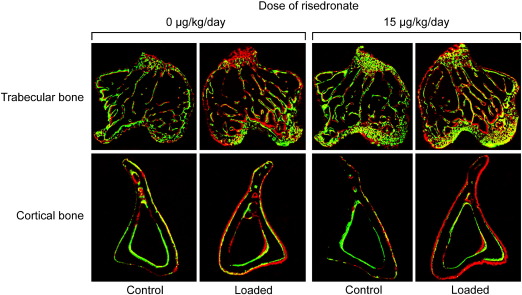
Representative transverse fluorochrome-labeled images of the left control and right loaded trabecular (approximately 0.25 mm distal to the growth plate) and cortical (approximately 37% of the bone's length from its proximal end) bone in the tibiae of mice treated with vehicle or risedronate at a dose of 15 μg/kg/day. Green: calcein label injected on the first day of loading (day 4). Red: alizarin label injected on the last day of loading (day 15).

Mechanical loading-related increases in trabecular BV/TV and cortical bone volume, as assessed by the difference between the right loaded tibiae and their contra-lateral non-loaded controls, were not significantly influenced by treatment with risedronate, even when given at a high dose (15 or 150 μg/kg/day) (Figs. 3 and 4). Consistent with previous reports [34,40], the fluorochrome-labeled images supported the inference that such loading-related bone gain was primarily associated with increased osteogenesis (Fig. 5). The additive effect of risedronate and loading on trabecular BV/TV was found at a dose of 15 or 150 μg/kg/day (Table 2, Fig. 2), while there was no synergistic effect of risedronate and loading on trabecular or cortical bone at any dose (Fig. 3). A slight reduction in the loading-related increase in trabecular thickness was observed with high doses of risedronate, but this only reached statistical significance at a dose of 15 μg/kg/day (Fig. 3).

## Discussion

In the present study, vehicle or risedronate at various doses was administered to 17–19 week old female C57BL/6 mice and changes in the structure of the tibiae three-dimensionally analyzed by high-resolution μCT. Although the treatment period was short, high doses of risedronate (15 and 150 μg/kg/day) resulted in higher trabecular BV/TV and trabecular number. This was expected as it has been established that treatment with bisphosphonates induces higher trabecular bone mass by suppressing bone resorption especially during the growing period [41]. In contrast, no significant changes in cortical bone volume were detected with risedronate treatment at any dose, while a low dose of risedronate (1.5 μg/kg/day) resulted in slightly lower periosteally enclosed volume. Some previous studies also found that risedronate treatment suppressed periosteal bone formation in intact mice [42] and rats [43], but no significant effects of risedronate on periosteal apposition were detected in skeletally mature ovariectomized rats [27,44] and dogs [45,46]. Taken together, these studies suggest that when the skeleton is no longer growing risedronate would have a negligible effect on the periosteal surface.

As validated previously [34], we assessed the effects of loading by comparing the architecture of the tibiae on one side, which received no artificial loading, with that on the contra-lateral side which was subjected to a regimen of non-invasive, dynamic axial loading sufficient to engender an osteogenic response. Consistent with previous studies [34,37,38], mechanical loading produced increases in both trabecular and cortical bone mass in all loaded limbs, primarily by increased trabecular thickness and periosteal expansion, respectively. Such loading-related bone gain was not reduced by treatment with risedronate, even when given at a very high dose (150 μg/kg/day). As a result, the effect of high-dose (15 or 150 μg/kg/day) risedronate and loading on bone mass was additive in the trabecular region. There was no synergistic effect of risedronate and loading on either trabecular or cortical bone at any dose. Although the loading-related increase in trabecular thickness was marginally reduced by risedronate at a dose of 15 μg/kg/day, this could be due to lower mechanical strains engendered resulting from the higher trabecular bone mass by the risedronate treatment.

These results are consistent with previous histomorphometric findings in the rat showing that the osteogenic response to mechanical stimulation is not altered by bisphosphonates [26,27]. In the first of these studies [26], the tail vertebrae were invasively loaded in the presence or absence of pamidronate and new bone formation induced by loading in the trabecular region was independent of bisphosphonate treatment. In the second [27], the effect of alendronate, risedronate and zoledronic acid at clinical doses on load-induced cortical modeling in the rat ulna was investigated following ovariectomy and none of these bisphosphonates significantly inhibited periosteal apposition. In contrast, a recent experiment using the mouse tibia suggested that there was a negative interaction between zoledronic acid and mechanical loading in cortical bone [28]. However, this could be associated with lower mechanical strains engendered resulting from the increased cortical bone mass by the zoledronic acid treatment.

It remains to be determined whether other anabolic therapies besides mechanical loading, when used in conjunction with an anti-resorptive agent, have a negative, additive or synergistic effect on the skeleton. Clinical evidence has shown that there can be a negative interaction between alendronate and intermittent parathyroid hormone, the only anabolic drug currently licensed for osteoporosis treatment [47,48]. This interaction seems to be less for risedronate than alendronate [49,50]. On the other hand, mechanical loading in rodents has been shown to suppress sclerostin expression in osteocytes [39,51], and sclerostin neutralizing monoclonal antibody also increases bone formation independently of bone resorption in humans as well as rats [52,53]. Further elucidation of the osteogenic pathways induced by mechanical loading will therefore offer the potential for developing potent anabolic approaches which can act independently of bone resorption.

In conclusion, mechanical loading-related increases in both trabecular and cortical bone mass are not reduced by even high doses of risedronate in almost skeletally mature female mice. This experimental evidence suggests that osteogenic exercise can have beneficial effects on bone health independently of those derived from the anti-resorptive effects of bisphosphonates in patients with osteoporosis.

## Figures and Tables

**Table 1:** Body weight and longitudinal lengths of the left control and right loaded tibiae in mice treated with vehicle or risedronate at a dose of 0.15, 1.5, 15 or 150 μg/kg/day.

Dose of risedronate (μg/kg/day)	0 (n = 20)	0.15 (n = 10)	1.5 (n = 10)	15 (n = 10)	150 (n = 10)
Body weight					
Initial (g)	23.2 ± 0.3	23.4 ± 0.4	23.1 ± 0.5	23.3 ± 0.6	23.0 ± 0.4
Final (g)	22.6 ± 0.2	22.9 ± 0.4	22.1 ± 0.4	22.5 ± 0.6	22.5 ± 0.5
Length of the tibia					
Left control (mm)	18.0 ± 0.1	18.0 ± 0.1	17.9 ± 0.1	18.0 ± 0.1	18.0 ± 0.1
Right loaded (mm)	18.1 ± 0.1	18.0 ± 0.1	18.0 ± 0.1	18.0 ± 0.1	18.0 ± 0.1

Mean ± SE. No significant differences between vehicle and risedronate at a dose of 0.15, 1.5, 15 or 150 μg/kg/day by one-way ANOVA.

**Table 2:** Trabecular and cortical μCT parameters in the left control and right loaded tibiae in mice treated with vehicle or risedronate at a dose of 0.15, 1.5, 15 or 150 μg/kg/day.

Dose of risedronate (μg/kg/day)	0 (n = 20)	0.15 (n = 10)	1.5 (n = 10)	15 (n = 10)	150 (n = 10)	*p* value^a^		
						Risedronate	Loading	Interaction
*Trabecular bone*								
Bone volume/tissue volume								
Left control (%)	16.6 ± 0.3	17.1 ± 0.4	17.7 ± 0.3	20.9 ± 0.6	21.3 ± 0.4	< 0.001	< 0.001	0.655
Right loaded (%)	22.7 ± 0.5	22.6 ± 0.5	23.2 ± 0.6	26.4 ± 0.7	27.7 ± 0.8			
Trabecular number								
Left control (mm^− 1^)	3.02 ± 0.05	3.07 ± 0.08	3.26 ± 0.06	3.66 ± 0.10	3.60 ± 0.08	< 0.001	< 0.001	0.697
Right loaded (mm^− 1^)	3.38 ± 0.05	3.40 ± 0.10	3.53 ± 0.08	4.03 ± 0.10	4.04 ± 0.13			
Trabecular thickness								
Left control (μm)	55.1 ± 0.5	55.7 ± 0.8	54.4 ± 0.6	57.3 ± 0.8	59.2 ± 0.9	0.009	< 0.001	0.011
Right loaded (μm)	67.1 ± 0.6	66.5 ± 0.9	65.8 ± 1.0	65.5 ± 0.8	68.6 ± 1.1			

*Cortical bone*								
Bone volume								
Left control (mm^3^)	0.380 ± 0.003	0.383 ± 0.003	0.366 ± 0.005	0.377 ± 0.007	0.372 ± 0.007	0.815	< 0.001	0.725
Right loaded (mm^3^)	0.481 ± 0.006	0.479 ± 0.006	0.476 ± 0.008	0.479 ± 0.009	0.481 ± 0.007			
Periosteally enclosed volume								
Left control (mm^3^)	0.627 ± 0.005	0.614 ± 0.006	0.594 ± 0.008	0.611 ± 0.011	0.607 ± 0.012	0.111	< 0.001	0.243
Right loaded (mm^3^)	0.717 ± 0.005	0.713 ± 0.008	0.709 ± 0.006	0.704 ± 0.010	0.709 ± 0.007			
Medullary volume								
Left control (mm^3^)	0.247 ± 0.003	0.231 ± 0.004	0.228 ± 0.005	0.234 ± 0.006	0.236 ± 0.007	0.096	0.085	0.030
Right loaded (mm^3^)	0.237 ± 0.004	0.234 ± 0.006	0.234 ± 0.005	0.226 ± 0.007	0.228 ± 0.005			

Mean ± SE.

a Mixed model analysis including body weight.
